# How is primary care nursing embedded in nursing undergraduate curricula: A mixed‐method study from four countries

**DOI:** 10.1002/jgf2.711

**Published:** 2024-08-22

**Authors:** Mayumi Kako, Elizabeth Halcomb, Mariko Mizukawa, Michiko Moriayama

**Affiliations:** ^1^ Division of Nursing Science, School of Biomedical and Health Sciences Hiroshima University Hiroshima Japan; ^2^ School of Nursing, Faculty of Science, Medicine & Health University of Wollongong Wollongong New South Wales Australia; ^3^ Kobe City College of Nursing Kobe Japan

**Keywords:** curriculum positioning, healthcare policy, nursing education, primary care nursing

## Abstract

**Introduction:**

Given the increase in the primary care nursing workforce and the need to further grow nursing roles in the community understanding how this is included in undergraduate education is important. This study aimed to explore the inclusion of primary care in the undergraduate nursing education curriculum of four countries.

**Method:**

A mixed‐method design was employed to obtain a broader context of primary care nursing in nursing education and teaching approaches relating to primary care nursing. Subsequently, Australia, Canada, Spain, and Ireland were selected for this study because primary healthcare systems are established as a part of their healthcare system.

**Results:**

In total, 136 nursing faculties (40 in Australia, 35 in Canada, 46 in Spain, and 15 in Ireland) were invited to participate in this study. Of these, 27 responses were obtained (19.8% response rate). Following the survey phase, in‐depth interviews were conducted with 13 participants. The results indicated that the highest number was coded within the theme of “Understanding of PHC in the curriculum” (*n* = 108). The second highest number (*n* = 87) was within the theme of “Interpretation differences of PHC in curriculum,” and the third highest (*n* = 31) was coded within “Policy impact on health by national government and others.”

**Conclusion:**

The results emphasized the ambiguity of primary care within the undergraduate nursing curriculum and that interpretation and implementation into the curriculum largely depended on the school's intentions.

## Graphical abstract

This manuscript presents the result of how primary care nursing was embedded into curricula. The mixed method was used to survey and interview from four countries including Australia, Canada, Ireland, and Spain.

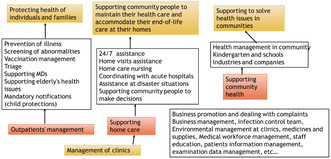

## 1 INTRODUCTION

Primary care is the first point of contact for patients with the healthcare system.^1^ Internationally, primary care services have grown and developed to meet growing community needs for care related to aging populations and the growth in chronic conditions. While the specific services vary internationally, there has been a growth in the multidisciplinary nature of primary care services. In particular, the number of nurses being employed in primary care has increased across the globe.^2^ In order for nurses to be prepared for primary care roles it is important to understand the preparation that they receive in their professional education.^3^, ^4^

This study was originally motivated by how primary care nursing is incorporated within nursing education in Japan. The current healthcare system in Japan is somewhat different from the international context in that while patients consult general practitioners, they can also access medical specialists directly.^5^ Although differences and similarities in healthcare systems exist between Japan and the countries included in this study, nurses are the second largest workforce in care systems, following the hospital setting.^6^

While nurses are the largest workforce in healthcare sector globally, nurses are also the largest co‐workers with physicians and other allied health professionals in primary care. Primary care has been facing the challenge to observe the decreasing workforce on the contrary increasing the demands of community in some coutries.^7^, ^8^ For example, according to the Ministry of Health, Labor and Welfare in Japan, nurses working in hospitals accounted for 69.0% (working in clinics and general practice; some clinics have admission beds), nurses in nursing care facilities accounted for 7.9%, and district nurses accounted for 4.9% in 2020.^6^ In Australia, general practice was the top place of employment for primary care nurses and counted 68% of nurses working in all primary care sector.^9^ In Canada, registered nurses counted approximately 70% of the primary care in community setting. Comparing to the increment of family physicians, the number of Nurse Practitioners who are qualified is steadily increasing.^10^

While the home visiting nurse workforce has been greatly emphasized over the last two decades because of the increasing older population in Japan, limited focus has been placed on nurses working in primary care who play unique roles, neither has it been recognized in nursing education.^10^

**FIGURE 1:**
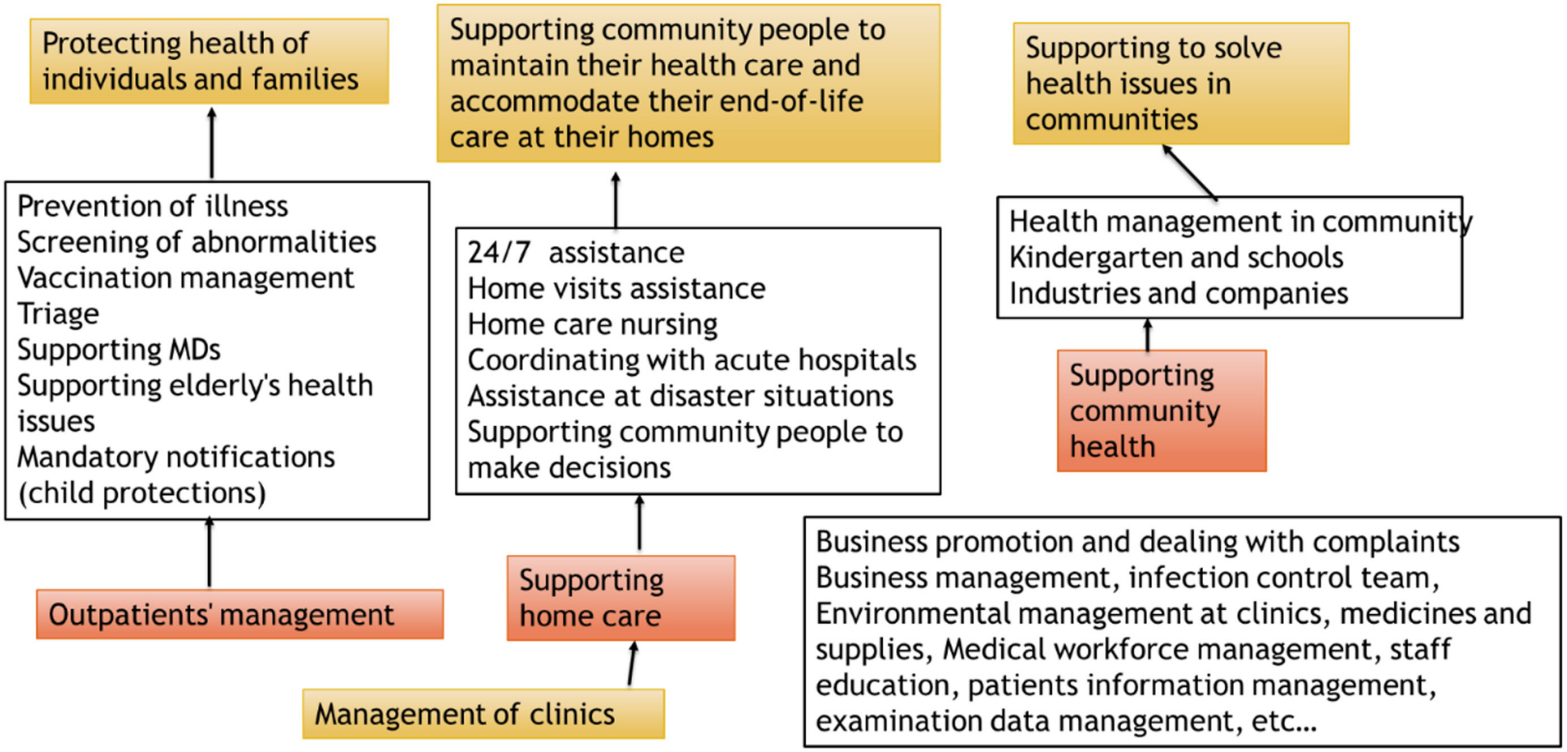
The role of nurses at GPs in Japan^5^ (originally in Japanese, translated by author).

Nurses working at clinics play unique roles; however, their role in Japan is not well recognized because of the traditional nursing role understood as the norm in smaller clinic facilities, as doctors' assistants.^11^ Figure 1 presents the complex and multiple roles of general practitioners.^12^ Further strengthen the nurse's role at clinics, the accreditation of those nurses was started in 2019 by the Japan Primary Care Association. However, primary care nursing is not established as one of the teaching subjects at nursing schools in Japan. Therefore, this study aimed to explore the question of how primary care nursing is informed and embedded in overseas nursing schools to establish how it can be implemented in Japan. The findings of this study will be beneficial for academics and nursing practitioners in Japan as well as overseas countries where the healthcare system is constantly under financial pressure; consequently, securing the nursing workforce.

The aim of this study is to explore the position of primary care nursing in undergraduate nursing education curricula in the selected countries. Understanding the position will be useful for nursing academics who are involved in curriculum development to make recommendations and raise awareness of this area of nursing.

## 2 METHODS

### 2.1 Study design

This study utilized a mixed‐method design to explore the inclusion of primary care in nursing education and teaching approaches relating to PCN.^13^ To achieve this, a cross‐sectional survey was conducted with nursing schools from the selected countries. Subsequently, semi‐structured interviews were conducted with academics to further explore the topic.^13^ The selection of this approach was to achieve the broader view of understanding of the concept of primary care in nursing curriculum first and to gain deeper understanding of academics toward the concept.

### 2.2 Sample

This study selected countries where primary care was established as a part of their healthcare system, namely Australia, Canada, Spain, and Ireland. Within those countries, universities with a school of nursing were selected as the samples to distribute the online surveys. In total, 136 nursing schools (Australia [*n* = 40], Canada [*n* = 35], Spain [*n* = 46], and Ireland [*n* = 15]) were selected from the official list by the school accreditation organizations website and surveys were disseminated to those schools. The potential participants for in‐depth interviewing were approached after the completion of the survey.

### 2.3 Data collection

An invitation email was directed to the administrator of each nursing school with a request to forward the email to the course coordinators for primary care nursing units. The request email contained the invitation letter, the study instructions, and the online survey link (Survey Monkey®). The survey contained the questions to ask the unit/subject numbers, the grade taught, the number of unit, the methods of assessment and the teaching mode. The survey was developed with the expert consultation who are familiar in this area of work. Once the survey was distributed, we sent the reminder 2 weeks prior to the closure date. The survey was conducted between October 2021 and October 2022.

Participants for in‐depth interviewing were recruited through the initial data collection. After completing the first survey, participants who wished to participate in the interviews were asked to provide their details to enable the researchers to contact them for the interviews. The survey results, particularly perspectives describing opinions on the importance of primary healthcare nursing in curricula, were used as interviewing guidance to inform and structure the subsequent interviews. Additionally, the overview of the survey results was used to promote communication between the participants during the interviews. The interviews were conducted online because of COVID‐19 travel restrictions. After the participants were given the study's description, the consent forms were signed and sent back to the researchers via email. The participants indicated a convenient time and agreed that the interview be audio‐recorded for analysis. All interviews were conducted in English and interviewing guidelines were utilized. The duration of each interview was between 45 and 75 min. Interviewing was conducted between December 2021 and October 2022.

### 2.4 Data analysis

The survey was imported into Excel® (Microsoft Corporation) for descriptive analysis. Descriptive statistical methods, such as frequencies, means and standard deviations were used to analyze data. Data was analyzed based on country and teaching methods. The open‐ended questions answers were transcribed and highlighted the comments of participants according to the questions.

Verbatim transcripts were created based on the audio‐recoded interview data, de‐identified and imported to NVivo. The collated data on NVivo was read through by researchers, codes were highlighted, and meaningful groups were made into themes.^14^, ^15^ The extracted themes were consulted with the team members to ensure the study's confirmability.^16^

### 2.5 Ethics approval

Approval was obtained from the Hiroshima University Epidemiology Ethics Review Committee (XXXX). Participation in this study was fully voluntary, and participants were allowed to withdraw at any time.

## 3 RESULTS

### 3.1 Quantitative findings

#### 3.1.1 Demographic

Invitations were sent to 136 nursing schools in four countries (40 in Australia, 35 in Canada, 46 in Spain, and 15 in Ireland). Of these, 27 responses were obtained (19.9% response rate), 15 (37.5% response rate) from Australia, 5 (14.3%) from Canada, 2 (13.3%) from Ireland, and 5 (10.9%) from Spain.

#### 3.1.2 Teaching year

The primary care nursing‐related topic was predominantly taught during the second and third years of the undergraduate program (35% and 33%, respectively). All countries, except Australia, offer a 4‐year nursing program, with variations concerning when the primary care nursing‐related topic is taught.

#### 3.1.3 The title of the PCN‐related topics

Table 1 presents the title of the PCN‐related unit/subject. The participants could indicate up to three units/subjects related to PCN in their affiliated schools. If unit/subject 1 was indicated, participants were asked to list the name of the unit/subject. Subsequently, if they had more than one PCN‐related subject, they were asked to list the second subject in the “Unit/subject 2” section.

**TABLE 1:** Subjects related to PCN.

Unit/subject 1 (*n* = 13)	
Nursing context of practice: Primary health care	Health and society
Community health nursing (*n* = 2)	Community and primary health nursing
Primary health care	Primary health care nursing
Primary Health Care 1 – Introduction to primary health care	Community and population health theory
Primary health and community nursing	Family and community nurse attention
Older persons and aging	Community Nursing 1 (epidemiology and community health)
Health, culture and society	

Unit/Subject 2 (*n* = 6)	
Global and national health	Primary Health Care 2 – Primary health care practice
Introduction to specialty practice: community health nursing	Community Nursing 2 (most prevalent diseases/communicable diseases/environmental health)
Indigenous culture and health	Development over the lifespan

Unit/Subject 3 (*n* = 5)	
First people's health	Community interventions
Nursing practice	Practice (clinical placement)
Legal, ethical, and professional practice issues	

The participants listed a total of 24 subjects. Among those, seven subjects were directly named PCN and five subjects were named community nursing. In the first list, more subjects with “PCN” and “community nursing,” contained in the name, were observed. The remaining subjects included topics such as family and older adult care, and health society. While this study did not investigate the syllabus of each unit/subject, it was assumed that the participants understood the subjects focusing on the topics of PCN within their schools. Interestingly, some participants listed subjects such as “older persons and aging” and “Development over the lifespan,” which can be focused on life course and health. Similarly, some participants listed subjects related to culture and society, such as “Health, Culture, and Society,” “Indigenous Culture and Health,” and “Legal, Ethical, and Professional Practice Issues.” The participants from Australia and Canada, where health disparities are reported in Indigenous communities, indicated that the concept of primary healthcare nursing is explicitly included in their curricula.

#### 3.1.4 Teaching methods

Teaching methods used for the unit/subject were predominantly workshops (31%), classroom lectures (22%), and clinical placement (20%). This was because of the difference in the topic structure and whether it included a teaching activity component such as focusing on the theoretical components or workshops focusing on the technical procedure acquirement. Some schools reported teaching unit/subjects that had only theoretical components, and some schools reported teaching unit/subjects that had both theoretical and technical components.

#### 3.1.5 Assessment methods

**FIGURE 2:**
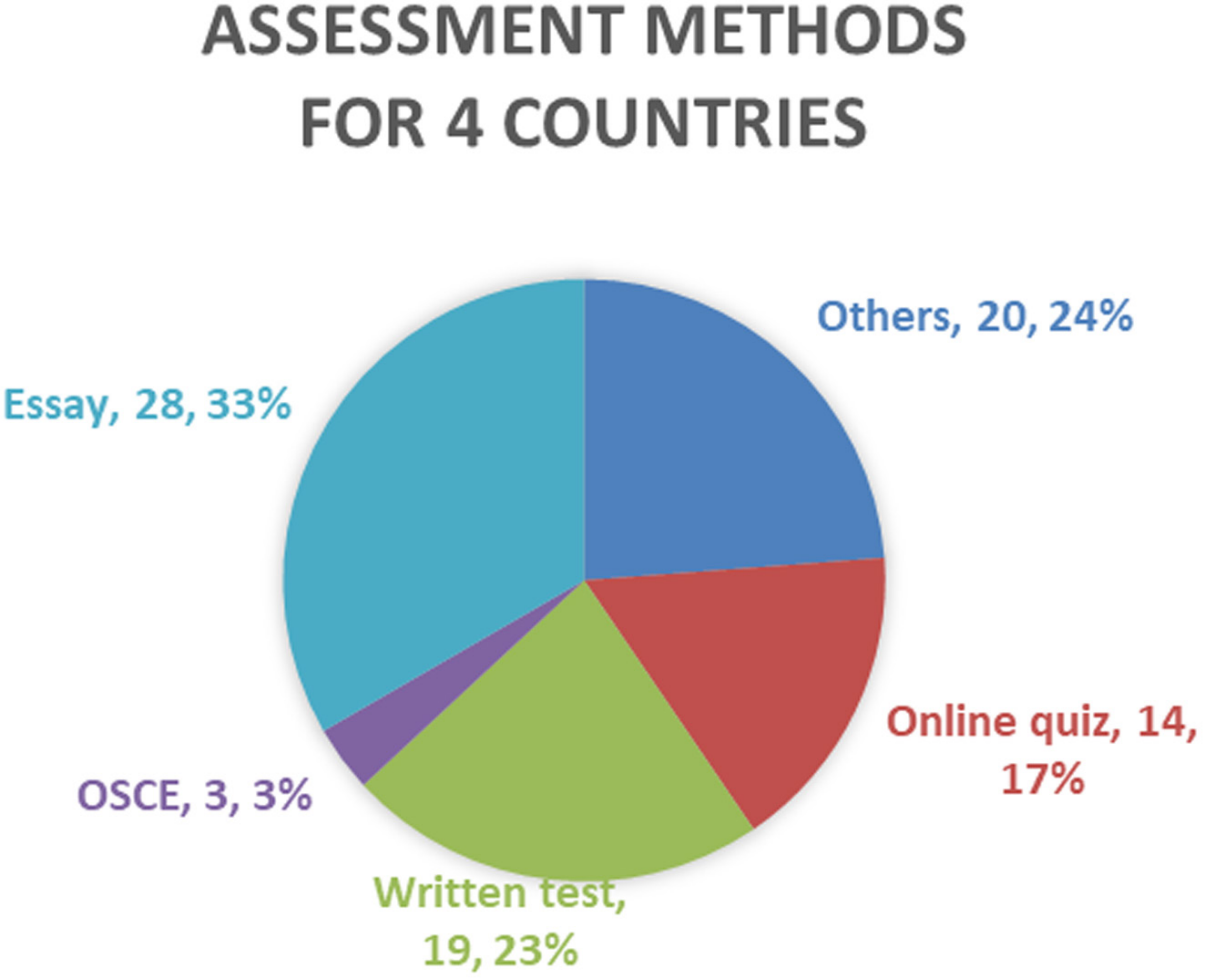
Assessment methods of the topic.

Figure 2 presents the unit/subject assessment methods. Writing essays was the most common type of assessment followed by written tests (23%) and online quizzes (17%). Since the survey was conducted during the COVID‐19 pandemic, some unit/subjects were being offered online and the assessment methods were changed to online‐based methods such as online quizzes. However, the teaching methods were predominantly flipped classrooms, including pre‐recorded lectures and tutorials, and workshops were either in‐person or online depending on each school's COVID‐19 situation.

#### 3.1.6 The importance of PCN

In the last section of the survey, participants were asked about the importance of PCN. Of the responses, 16 of the 17 described PCN as “very important.” The reasons for the indicated importance of PCN were described as follows by participants:

- “Primary Health care underpins nursing care and health.”
- “[Primary health care is] Integral to comprehensive nursing practice in partnership with clients.”
- “It is essential for the sustainability of the health care system that nurses understand and have the skills to practice in primary care.”
- “It is important since every nurse should know the importance of primary care settings and how to develop their work in primary care centers, implementing a perspective beyond individual care.”

Answers on its invisibility as a subject in the nursing curriculum were also reported.

- Primary care nursing is one of those specialties that is undervalued, and its importance is not realized to its full extent. There needs to be a better understanding among nurses regarding primary health care.
- We do not have many discrete subjects on primary care nursing since the content is addressed in many subjects as part of the patient journey.

### 3.2 Qualitative findings

Thirteen participants participated in the in‐depth interviews, 10 female and three males (Table 2). All participants were licensed nurses and faculty members possessing undergraduate or graduate experience in primary (health) care or community nursing.

**TABLE 2:** Interview participants.

Country	No. of participants	Gender
Australia	9	7 females, 2 males
Canada	2	2 females
Ireland	1	1 female
Spain	1	1 male
Total	13	10 females, 3 males

#### 3.2.1 Themes and description

Table 3 presents the interview analysis results. The highest coded number was within the theme of “understanding of PHC in the curriculum” (*n* = 108). The second highest number (*n* = 87) was within the theme of “interpretation differences of PHC in curriculum.” The third highest (*n* = 31) was coded within “Policy impact on health by national government and others.”

**TABLE 3:** Extracted themes.

Themes	Sub‐categories	Number of files cited	Number of code
Understanding of PHC in the curriculum		10	108
	Curriculum inclusion of PHC	5	21
	Scope of practice	4	7
Interpretation differences of PHC in curriculum		11	87
Policy impact on health by national government and others		9	31
COVID‐19 and its impact on community nursing		6	16
National health trend and primary care		5	5
Collaboration between PHC and NPs		1	2
	Complexity of post graduate course	1	1
Considering PHC as a career option		1	2
Graduate students' attributes (Competencies)		1	2
Economic impact brought by PHC		1	1
Relationship with other certifications		1	1

#### 3.2.2 Understanding of primary health in the curriculum

Participants most talked about their understanding of the importance of primary care to national health priorities, which also supports the curriculum positioning. One of the participants from Ireland stated:

What we have in our curriculum is very much in line with the strategic vision for health in our country, and fairness and inequality. (Ireland 1)

Participant A highlighted how the national health strategy impacts their curriculum, and that it needs to be supported by the principle of PHC. Furthermore, a participant from Australia discussed how the community‐focused curriculum needs to be embedded into the curriculum while noting that the principle of PHC is widely understood.

…the things like consumer participation and Person‐Centered Care, all of those things that are embedded now in practice is fairly standard, no matter where you are practicing, really steal from primary health care ideology… An increased emphasis on care outside of hospital settings outside of the acute setting… I suppose that that is really how it is woven through. It is supposed to be a full principle that runs throughout the curriculum. (Australia 3)

A participant from Canada further added to the role of nurses in this space of primary health care as follows:

I would like there to be more of an emphasis on community, the importance of community to make changes because, at the individual level, you can make changes for yourself. But when you start impacting the community, there is more societal impact. And so, there is phenomenal empowerment and advocacy that we could do as nurses for sure. (Canada 1)

#### 3.2.3 Interpretation differences of PHC in curriculum

While some schools did not have primary care nursing embedded into their nursing curriculum, a participant from Australia stated:

There is not explicitly a primary health care unit. However, there is a unit on healthy aging, which would, you know, talk about principles that are drawn from primary health care, philosophy, and ideology… (Australia 8)

Another participant described how primary care nursing was dealt with in their curriculum as concepts woven through their curriculum. The participant from Canada described this as follows:

So, for us here, we have a concept‐based curriculum. Primary health care is one of the concepts along with, you know, health promotion, social justice, and health equity. So, those are expected to be threaded throughout the curriculum. (Canada 2)

#### 3.2.4 Policy impact on health by national government and others

Participant A from Australia compared her nursing education experience in the 1980s with the current education, which is influenced by the national health policy as follows:

There have been some significant shifts in policy with our government. At the moment, they have just closed the primary healthcare ten‐year plan consultation draft paper. So, looking at the Australian primary healthcare system, as a health system in its entirety as a service model…that is very much, I feel, linking to the WHO's operational framework that they released in 2020…And I think with the changing of service models by government, and the policies that are changing, the diversity for nurses now, compared to potentially when I started nursing in the early 80s, is so different. (Australia 9)

Another participant from Australia emphasized the change and its impact on the curriculum at her school:

I would say there has been a lot of change. There has been a lot more recognition of the importance of primary healthcare in the last decade or so. And certainly in our curriculum, at the moment we are rewriting or we are refreshing our curriculum… In the new curriculum, we are hoping to have elements of primary healthcare in all subjects. So it is a constant education about primary healthcare throughout most of the subjects. There will still be a separate primary healthcare‐focused clinical placement, but concepts of primary healthcare, health prevention, patient education, and so forth will be more prevalent in all of the subjects… (Australia 10)

However, there are some differences, depending on the country, on how primary health care is implemented as a part of their healthcare system. A participant from Ireland stated:

…they [primary health care] are slower and taking off. Because we have got our GP service, our Doctor Service has always been either private or within the health system. So it is very difficult to bring somebody from their own private practice into more health service‐led primary health care centers. So, the primary health care centers, how are they being set up, this is also in its infancy, it is taking time, it is very slow. But in those primary health care centers, you have all of the multidisciplinary team members together, being available together, and it is a hub in the community. So, we are moving a lot of our facilities from the acute hospital setting into the community (Ireland 1)

All interviewed countries utilize the primary healthcare system as a part of their national healthcare system while the utilization of the system varies.

## 4 DISCUSSION

This study included participants from countries where PHC is implemented. The quantitative results indicated various teaching areas in PCN in different grades during the undergraduate course. This was assumed to be because of the topics covering PHC being broad, depending on the objectives of the subject. Furthermore, curricula were designed in consideration of their compatibility and integration with surrounding subjects. For example, some participants described subjects focusing on cultural nursing and older adults within the scope of PCN. This finding is supported by Lukewich et al.'s (2023) study.^17^ Their study found that undergraduate nursing students in Canada were expected to cover all competency areas while a gap exists regarding the contents of primary care included in undergraduate nursing competencies.^18^ The exposure to the topic may impact the student's perception of nursing and may influence their career choices.^19^, ^20^, ^21^

Based on the qualitative interview findings, some participants understood PHC as a philosophy and an approach to the community and the target population. The terms primary care nursing and primary healthcare nursing are often used interchangeably, which may be one of the reasons why PHC was viewed as a part of the philosophy curriculum; because of its nature and definition. In Japan, PCN and PHC are often used interchangeably, which should be considered when developing curricula. Moreover, consensus is required concerning established areas of nursing such as community nursing and public health nursing, which overlap the scope of practice in the context of nursing in Japan. For example, the competency standard in primary care was published by the Canadian Family Practice Nurses Association,^18^ and the national practice standards by the Australian Nursing and Midwifery Federation.^22^ Other participating countries, such as Spain, emphasized the importance of the primary care role during the COVID‐19 pandemic,^23^ and the advanced nursing role, with higher autonomy, while working in the community was highlighted. Regarding the Japanese context, primary care's impact on hospitalization was investigated and a positive association was found between high‐quality primary care and less hospitalization.^24^ Although this study focused on primary care from medical officers' perspectives, nurses are co‐working with them. While the primary care systems are beginning to be fully implemented after a long period of partial implementation in Ireland, the economic benefits of such care are beginning to be examined.^25^

It is difficult to compare and contrast the different maturity stages of primary care system implementation in each country; however, nurses work as a part of the team. Along with each healthcare policy development, it will be essential to be explicit regarding the effectiveness and quality of primary care nursing.

This study focused on four countries where the primary healthcare system was part of their country's healthcare system; consequently, the data in this study may have been skewed. The all‐surveyed country has primary care system embedded in their healthcare system; therefore, studying this topic is accepted as usual topic. Additionally, the quantitative data obtained in this study did not adequately represent all of the examined countries. The authors were aware of the different countries' healthcare systems and to minimize the biased perspective, the researchers informed each other to seek consensus to add further descriptions and understanding and avoid biased interpretations.

Based on the result of this study, followings are recommended:

- Defining clearly primary care nursing and their roles;
- Mapping how primary care nursing to be fit into the current nursing curriculum;
- Expanding the collaboration between education arena and clinical setting to provide educational opportunities for nursing students to promote primary care nursing.

## 5 CONCLUSION

This study explored how PHC was incorporated into the curricula of the targeted four countries. All these countries have established primary healthcare systems and nurses' role and scope of practice was publicized. However, the topics related to PHC are ambiguous, depending on the school's interpretation of PHC in the curriculum. Community nursing and care shift has been occurring over the last two decades from hospital nursing and care in Japan. With the discussion of the home doctor promotion and the current national health policy, primary care nursing in Japan will be playing an indispensable role concerning how and what is taught to undergraduates, which requires further discussion and organization. Further investigation on how PCN can be presented in nursing education curriculum in Japan is expected in the future.

## FUNDING INFORMATION

KAKEN KIBAN(C) 21K11081.

## CONFLICT OF INTEREST STATEMENT

The authors have stated explicitly that there are no conflicts of interest in connection with this article.

## ETHICS STATEMENT

Ethics Approval Statement: Yes.

Patient Consent Statement: Yes.

Clinical Trial Registration: E‐2609.

## Data Availability

Yes, upon request.
